# Multimorbidity and mortality trends in the COVID19 syndemic in Madagascar covering 180 diseases 2015 2024

**DOI:** 10.1016/j.isci.2025.114444

**Published:** 2025-12-17

**Authors:** Modeste Kameni Nematchoua, Diana Ratsiambakaina, Faratiana Jenny Rasoariseheno, Rija Onintsoa Andriamasinoro, Nivoarimelina Zoly Rakotomalala, Razafindramboho Samoelà Hérédia, Nirina Henintsoa Raveloharimino, Herisitraka Raotoson, Harimbola Fiononantsoa Razaiarilala Rakotovazaha, Alphonsine Mboty Reziky, Rakotoson Mariette, Raphaël Fidelis Randrianarivo, Vohangy Marie Anita Randriamihaja, Hery Henintsoa Randrianirina, Pâquerette Voahirantsoa Razanamiarana, Joseph Michel Razafimahenina, Wabo Gilles Cédric, Gaël Lauricia Lalanirina, Lindsay Kouatie Njonger, Philippe Manjakasoa Randriantsoa, Luc Narda Randrianahasina, Rakotomalala Vololoniana Razafimanalina, Assoumacou Noro Flavia, Larissa Lalatina Randriamialy, Andriarimanana Hery Nirina Rakotoarisoa, Lethicia Lydia Yasmine, Zely Arivelo Randriamanantany

**Affiliations:** 1 School of Doctorate, University of Antsiranana, Antsiranana, Madagascar; 2 Energy and Environment Laboratory, Department of Physics, Faculty of Science, University of Yaounde 1, Yaounde, Cameroon; 3 National Institute of Public and Community Health, Ministry of Public Health, Antananarivo, Madagascar; 4 Institute of Tropical Odonto-Stomatology of Madagascar (IOSTM), University of Mahajanga, Mahajanga, Madagascar; 5 Faculty of Medicine of Mahajanga, Madagascar and CHU PZAGA Androva, Mahajanga, Madagascar; 6 Nutrition-Environment-Health Doctoral School of the University of Mahajanga, Mahajanga, Madagascar; 7 Faculty of EGS Antananarivo, Department of Sociology, Antananarivo, Madagascar; 8 Health and Demographic Statistics Department, Directorate of Studies, Planning, and Information System, Ministry of Public Health, Antananarivo, Madagascar; 9 University of Antananarivo, Faculty of Sciences, Department of Physics – High Energy Physics, Antananarivo, Madagascar; 10 Genius Processes and the System Industrial Agricultural and Food (GPSIAA) Doctoral School of the University of Antananarivo, Antananarivo, Madagascar; 11 Ecole Doctorale Sciences Humaines et Sociales, Université d’Antananarivo, Antananarivo, Madagascar; 12 Ministry of Public Health, Antananarivo, Madagascar; 13 Faculty of Medicine - University of Antsiranana, Antsiranana, Madagascar

**Keywords:** Health sciences, Public health, Environmental health

## Abstract

Multimorbidity and mortality shape population health in Madagascar, where chronic and infectious diseases interact across regions. Using surveillance data covering 180 diseases across 22 regions from 2015 to 2024, we quantified geographic disparities, dominant contributors to disease burden, and shifts associated with the COVID-19 syndemic. Stroke was the leading cause of death, with regional case-fatality rates often exceeding 45% and reaching nearly 99% for cardiac arrest in some urban areas. Stroke also showed strong correlations with hypertension (r = 0.97) and diarrhea (r = 0.98), highlighting interconnected vulnerabilities. During the COVID-19 period, overall disease frequency increased by 6.12% on average, though responses varied widely across conditions. Post-lockdown, incidence declined substantially (MRD = −90.17%), and mortality decreased slightly (MRD = −3.93%), yet disparities persisted. Groups with cardiovascular and mental health disorders exhibited the greatest instability. These findings illustrate how interacting epidemics reshape population vulnerability and underscore the importance of integrated strategies to strengthen health-system resilience.

## Highlights

• Stroke is the leading cause of death with extreme regional disparities
• Strong syndemic links found between stroke, hypertension, and diarrhea
• COVID-19 period shows a 6.12% rise in overall disease frequency
• Post-COVID decline in incidence, but major regional health inequalities persist

## Graphical abstract

**Figure:**
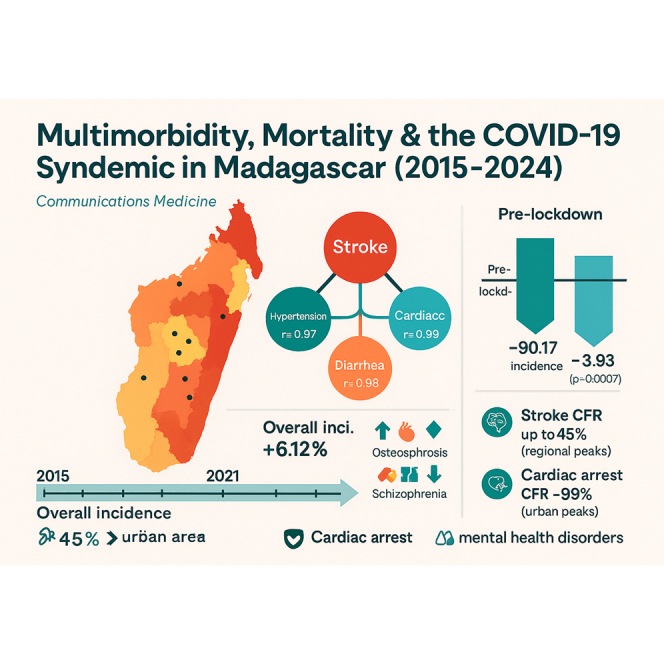

## Introduction

Coronaviruses, belonging to the *Coronaviridae* family, were first identified in humans in the 1960s^1^ In late 2019, the emergence of the novel coronavirus SARS-CoV-2 triggered the COVID-19 pandemic, causing unprecedented global health, social, and economic disruptions.^2^ In Africa, the impacts were multidimensional: 28% of economic actors in Algeria’s commercial sector were affected, 48% of households reported income loss, and over 36% faced food access difficulties. Similar patterns were observed in Madagascar, where the pandemic amplified structural vulnerabilities. Beyond a 2.2% estimated mortality rate as of July 2021, it strained the health system, disrupted supply chains, and deepened poverty and inequality, threatening national development progress.^3^ Prior to COVID-19, communicable diseases already dominated Madagascar’s health landscape. The 2017 plague outbreak caused over 2,400 suspected cases—77% pneumonic—and 200 deaths.^4^ Malaria remained a major concern, with 1.5 million reported cases in 2018, mainly in the west and south.^5^^,^^6^ Tuberculosis was endemic with 30,000–35,000 new cases yearly, including multidrug-resistant strains.^7^ Diarrheal and acute respiratory infections, exacerbated by malnutrition and poor water access, were leading causes of child mortality.^8^

Since 2019, COVID-19 has revealed its multisystemic nature. Initially considered a respiratory illness, it affects multiple organs through inflammatory pathways and ACE2 receptor distribution, with cardiovascular, renal, gastrointestinal, neurological, and dermatological manifestations, Gupta et al.^9^ Thromboembolic events among hospitalized patients prompted the inclusion of anticoagulants in treatment.^10^^,^^11^ Severe cases are linked to age, comorbidities, and excessive immune response (“cytokine storm”).^12^^,^^13^ The phenomenon of “long COVID” further illustrated the disease’s chronic dimension, characterized by persistent fatigue, respiratory and cognitive symptoms, and psychosocial impacts requiring multidisciplinary care.^14^^,^^15^ Recognized globally since 2022, long COVID prompted the WHO to establish guidelines for its management, emphasizing post-epidemic surveillance and patient-centered care. Following the pandemic, Madagascar faced both resurgent and emerging health threats, exposing systemic fragilities. Between 2022 and 2024, malaria remained the leading cause of outpatient visits and hospital deaths, with 2.8 million cases in 2023—particularly in southern and western regions affected by droughts and cyclones.^16^ Acute malnutrition persisted, affecting 358,000 children aged 6–59 months, including 83,000 severe cases (IPC Madagascar, 2024).^17^ Diarrheal diseases remained widespread, linked to unsafe water and poor sanitation (Rakotondramboa et al.).^18^ In 2024, intestinal parasitosis affected over 60% of school-aged children in Ambatoboeny (Razanajatovo et al.).^19^ Epidemic-prone diseases such as plague also persisted, with 200–400 annual cases and localized outbreaks, including five deaths in Ambohidratrimo.^20^

Non-communicable diseases have concurrently risen. Hypertension now affects nearly 37% of adults aged 30–79 years,^21^ while HIV/AIDS prevalence among high-risk groups is estimated between 9% and 25% (Randrianarisoa et al.).^22^ This double burden—infectious and chronic—demands renewed national priorities for equitable access to essential services and resilient health systems. This retrospective, cross-sectional, quantitative study analyzes routine hospital data to provide the first integrated, multi-year assessment of disease incidence, lethality, and multimorbidity patterns across Madagascar during the pre-pandemic (2016–2018), pandemic (2019–2021), and post-pandemic (2022–2024) phases. Specifically, it aims to: (i) quantify temporal and spatial variations in 180 diseases, (ii) examine syndemic interactions between infectious and non-communicable diseases, and (iii) evaluate pandemic-related impacts on morbidity and mortality. Applying a syndemic framework to regional surveillance data, this research generates evidence to guide targeted, multi-sectoral health strategies. Unlike prior disease-specific studies, it offers a comprehensive, longitudinal perspective essential for strengthening national health resilience.

## Results

### Evolution of data completeness and promptness in Madagascar’s regions

To control for potential temporal and regional fluctuations in reporting rates that could confound the results, we applied several measures during analysis. First, data completeness and promptness rates were calculated as annual averages per region, thereby minimizing the influence of short-term or seasonal variations. Second, year-to-year comparisons were performed using both Pearson and Spearman correlation analyses to assess the existence and strength of temporal trends, independent of irregular peaks or drops in specific months. Third, regional variability was addressed by systematically comparing performance within each region across the study period, rather than pooling raw figures, which could mask local inconsistencies. Outlier cases—such as regions recording extremely high or low rates—were retained in the analysis but highlighted separately in the interpretation to avoid skewing the national trend. This approach ensured that observed patterns reflected structural and programmatic dynamics rather than short-term seasonal effects or isolated administrative disruptions. Table 1 shows Completeness and Promptness percentage between 2016 and 2021 in the different regions. Completion rates vary considerably across regions and years. In 2016, the national average was relatively low (60.43%), indicating an incomplete implementation of the program. This situation improved significantly in 2017 (85.85%) and remained high in 2018 (84.08%) and 2019 (85.69%). However, a slight decline occurred from 2020 (74.05%) and worsened in 2021 (69.73%), which may be attributed to contextual factors such as health crises or local instability. From a statistical perspective, a Pearson correlation was calculated between the years (2016–2021) and the annual average completeness. The result shows a very weak correlation coefficient (r = 0.065) and a *p*-value of 0.902, suggesting that there is no statistically significant temporal trend. While fluctuations exist, they do not follow a linear or consistent progression over time. Some regions, such as Amoron’i Mania, Betsiboka, and Vatovavy Fitovinany, consistently achieved 100% completion, reflecting strong institutional commitment and efficient monitoring systems. In contrast, regions such as Melaky, Analanjirofo, or Androy displayed highly variable or declining completeness rates, with Melaky dropping from 91.7% in 2016 to 0% in 2021, highlighting sustainability or follow-up challenges in program implementation. In addition, Spearman’s correlation (r_s_ = −0.086) shows a very weak negative relationship between the year and the average completion rate, and this relationship is not statistically significant (*p* > 0.05). In other words, over the period 2016–2021, there is no significant linear trend toward a decrease or an increase in the completion rate at the national level, even if some fluctuations are visually present.

**Table 1:** Completeness and Promptness frequency between 2016 and 2021 in the different regions

	Completeness rate (%)						Promptness rate (%)					
Regions	2016	2017	2018	2019	2020	2021	2016	2017	2018	2019	2020	2021
Alaotra Mangoro	67.7	100	68.8	55.6	100	100	32.3	0	11.1	11.8	75	91.7
Amoron’i Mania	100	100	100	100	100	100	53	100	25	25	41.7	25
Analamanga	41.7	81.2	100	100	81.2	95.6	58.3	18.8	41.7	50	25.7	49.71
Analanjirofo	71.4	100	68.6	62.9	75	0	28.6	100	37.1	25.7	75	0
Androy	5.6	100	69.4	33.3	100	100	94.4	25	69.4	33.3	50	41.7
Anosy	25	83.3	100	91.7	41.7	83.3	75	8.3	100	83.3	16.7	
Atsimo Andrefana	8.3	95.8	91.7	100	83.3	92.5	91.7	50	41.7	75	33.3	58
Atsimo Atsinanana	58	100	100	100	66.7	0	100	100	16.7	33.3	16.7	0
Atsinanana	58.3	45.8	100	100	29.2	83.3	41.7	0	41.7	50	8.3	16.7
Betsiboka	75	100	100	100	100	100	25	0	100	75	25	80
Boeny	83.3	33.3	83.3	100	33.3	100	16.7	33.3	16.7	33	11.1	
Bongolava	58	100	47.2	33.3	100	100	100	8.3	30.6	8.3	66.7	66.7
Diana	50	30.6	100	100	33.3	100	50	30.6	0	66.7	33.3	91.7
Haute Matsiatra	58.3	100	0	100	91.7	41.7	41.7	58.3	0	100	58.3	8.3
Ihorombe	58	91.7	100	100	100	100	100	8.3	100	100	91.7	90
Itasy	37.5	100	45.8	50	100	100	62.5	41.7	12.5	0	25	11
Melaky	91.7	100	100	100	25	0	8.3	100	0	0	25	0
Menabe	66.7	100	91.7	100	66.7	0	33.3	8.3	91.7	100	8.3	0
Sava	83.3	100	100	91.7	41.7	0	16.7	33.3	54.2	50	16.7	0
Sofia	56.6	58.3	100	100	91.7	83.3	43.4	58.3	33.3	41.7	83.3	25
Vakinankaratra	67.7	68.6	83.3	66.7	68.6	54.3	32.3	45.7	8.3	16.7	22.9	2.9
Vatovavy Fitovinany	100	100	100	100	100	100	53	0	33.3	58.3	0	75

The average promptitude rate in 2016 stood at 52.87%, indicating relatively good responsiveness in delivering data or implementing actions. However, this dropped significantly in 2017 (37.39%) and remained consistently low, fluctuating between 36% and 46%, ending at 36.67% in 2021. Several regions, such as Melaky, Vatovavy Fitovinany, Menabe, and Analanjirofo, recorded a dramatic drop to 0% in 2021, reflecting an almost complete lack of promptitude. In contrast, regions such as Ihorombe and Diana maintained high performances (90% and 91.7% in 2021, respectively), pointing to better administrative coordination and follow-up. Statistically, the Pearson correlation coefficient between year and mean promptitude was r = −0.611, indicating a moderate negative correlation: as time progresses, promptitude tends to decrease. However, the *p*-value of 0.197 suggests that this trend is not statistically significant at the 5% level, though it does highlight a worrisome tendency over time. This decline may result from factors such as institutional fatigue, loss of motivation, shifting priorities, or external crises hampering the timely delivery of required actions or information.

### Regional distribution and lethality of major diseases in Madagascar (2015–2022)

**Figure 1:**
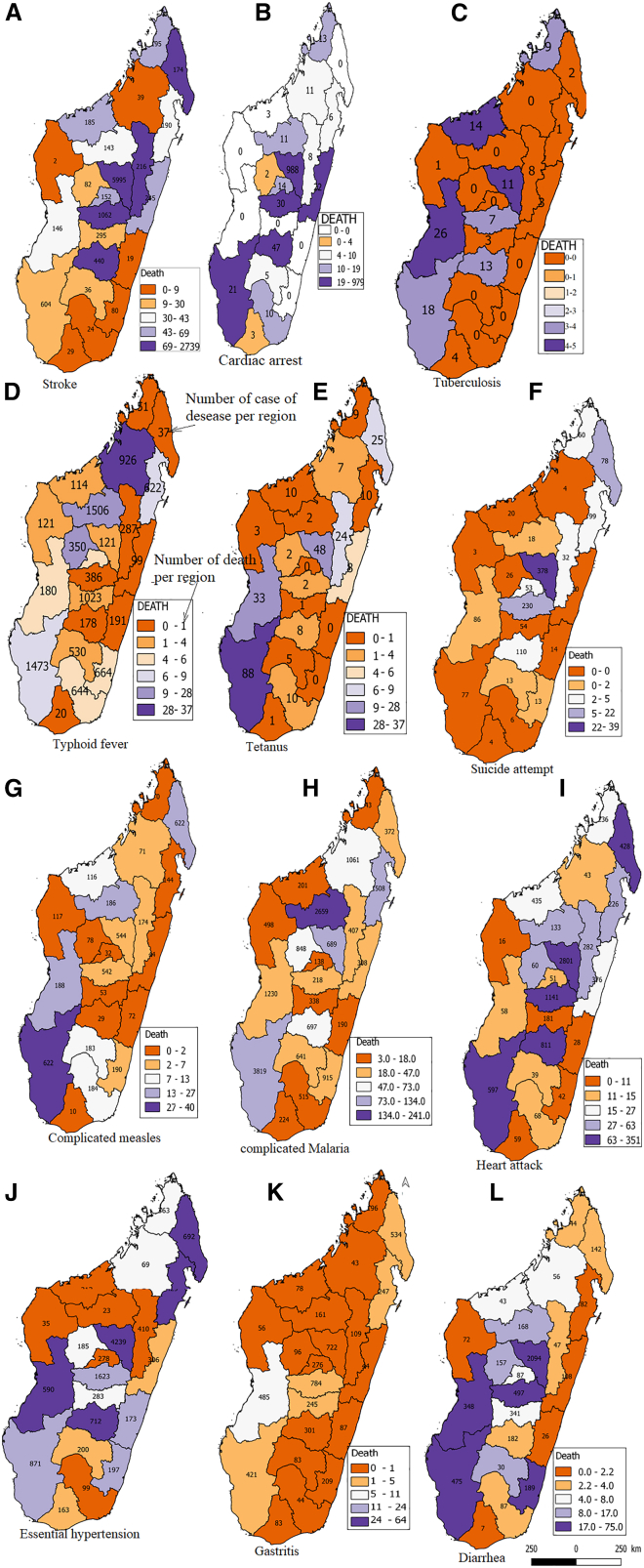
Average number of cases of disease and death per region between 2015 and 2022

Figure 1 shows the number of cases of disease and death per region between 2015 and 2022. The analysis of morbidity and mortality data from 22 regions of Madagascar (only case of 11 diseases, see Figure 1) reveals deep regional disparities and strong epidemiological interrelations between diseases. On average, the most prevalent conditions include Malaria (796 cases), hypertension (557 cases), stroke (471 cases), gastritis (241 cases), and diarrhea (240 cases), highlighting the dual burden of infectious and non-communicable diseases in the population. Stroke stands out as the most critical health indicator, not only due to its high incidence but especially its lethality, with an average of 170 deaths per region, the highest among all reported conditions. The capital region, Analamanga, followed by Vakinankaratra and Sava, accounts for over half of the total stroke-related deaths, possibly reflecting urban health inequalities, aging populations, or suboptimal management of chronic conditions. Correlation analyses show extremely strong and statistically significant associations between stroke cases and other cardiovascular and systemic conditions, such as cardiac arrest (r = 0.99, *p* < 0.00001), hypertension (r = 0.97, *p* < 0.000001), and heart failure (r = 0.95, *p* < 0.000001). This reinforces the idea of compounding risk factors within the cardiovascular spectrum. The high correlation with diarrheal diseases (r = 0.98, *p* < 0.000001) might be explained by systemic vulnerabilities, including dehydration, electrolyte imbalances, or lack of primary health infrastructure. Interestingly, stroke is also highly correlated with suicide rates (r = 0.89, *p* < 0.000001), suggesting a psycho-social burden linked to chronic illness and health despair in certain areas. Moderate but statistically relevant correlations were also identified with gastritis (r = 0.61), complicated measles (r = 0.49), and tetanus (r = 0.42), pointing to the role of multi-morbidity in exacerbating health outcomes. In contrast, conditions such as malaria (r = 0.009), typhoid (r = −0.13), and tuberculosis (r = 0.24) showed no significant correlations with stroke incidence, likely due to different epidemiological patterns, better disease management, or geographic divergence. Altogether, these findings confirm that stroke is not an isolated phenomenon but rather a complex health outcome emerging from intertwined chronic, infectious, and socio-behavioral drivers. A multisectoral response—combining cardiovascular care, mental health support, and public health reinforcement—is urgently needed to reduce stroke-related mortality across Madagascar.

**Figure 2:**
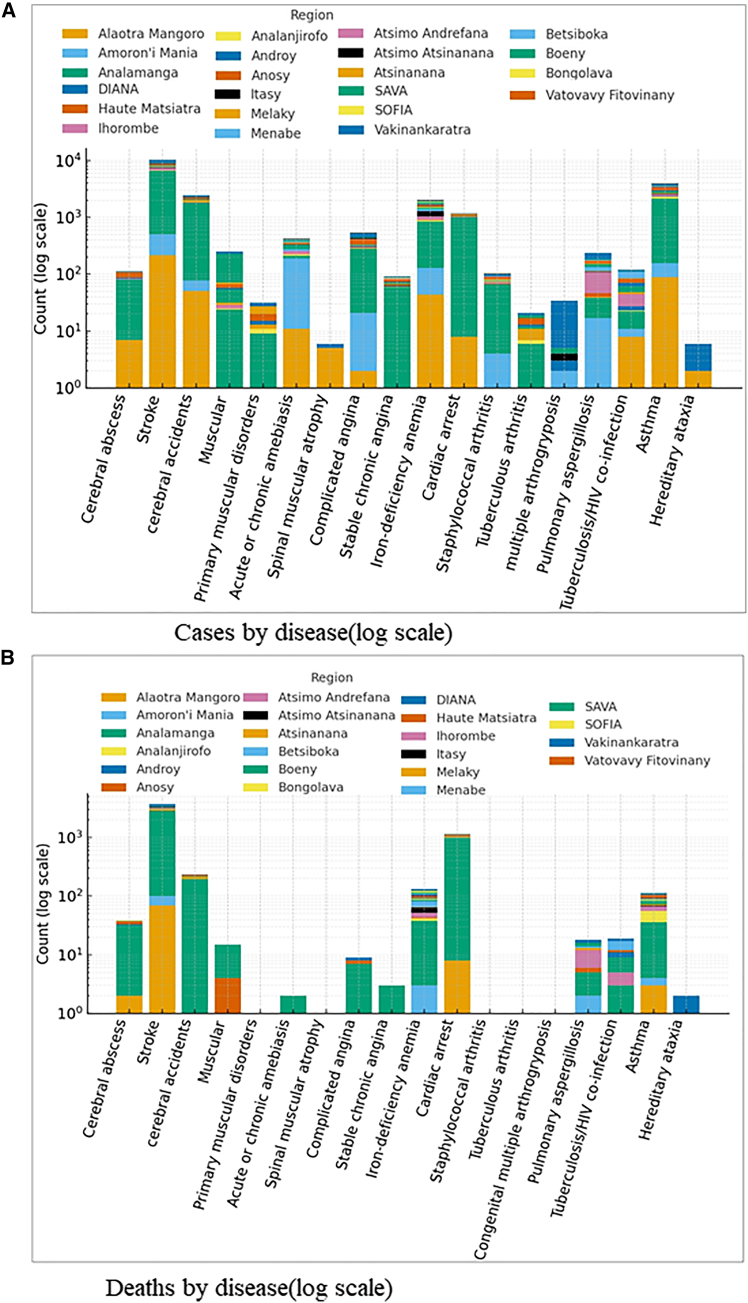
Regional distribution of disease cases and deaths in Madagascar (A) Randomly selected examples of disease cases per region between 2015 and 2022, drawn from the 180 diseases investigated across Madagascar. Each bar represents the total number of reported cases for a given disease, aggregated by region. The stacked colors indicate the regional contribution to the total number of cases. A logarithmic scale is used to facilitate comparison across diseases with markedly different incidence levels. (B) Randomly selected examples of deaths by disease per region between 2015 and 2022 among the same set of diseases. Bars represent the cumulative number of deaths associated with each disease, with colored segments denoting regional contributions. The logarithmic scale highlights the variability in mortality levels across diseases and regions.

As showed into Figure 2A, the analysis of epidemiological data from Madagascar’s 22 regions reveals stark disparities in disease distribution. On average, strokes are the most reported condition (mean ≈272 cases), followed by respiratory allergies and asthma (mean ≈120 cases), and iron-deficiency anemia (mean ≈70 cases). The Analamanga region stands out with extremely high case counts—5995 strokes, 988 cardiac arrests, and 716 cases of anemia—reflecting both high population density and greater access to diagnostic services. A strong and statistically significant correlation is observed between stroke and cardiac arrest (r = 0.88; *p* < 0.01), as well as between tuberculosis/HIV co-infection and iron-deficiency anemia (r = 0.74; *p* < 0.05), indicating common comorbidities or shared risk factors. In contrast, regions such as Androy, Anosy, and Atsimo Atsinanana report very low figures, likely due to limited healthcare access or underreporting. These findings highlight the urgent need to strengthen disease surveillance, decentralize healthcare infrastructure, and prioritize both chronic and infectious diseases in public health policies.

The analysis of disease-related deaths (see Figure 2B) reveals trends similar to those observed in case incidence, yet with even more pronounced regional disparities. On average, strokes (≈190 deaths) account for the highest number of fatalities, followed distantly by cardiac arrest (≈55 deaths) and respiratory complications such as asthma and allergies (≈6 deaths). The Analamanga region alone records nearly half of all stroke-related deaths (2739 cases) and the vast majority of cardiac arrest deaths (979), likely due to higher population density, improved reporting mechanisms, or increased prevalence of cardiovascular risk factors. A strong and statistically significant correlation is found between stroke and cardiac arrest deaths (r = 0.91; *p* < 0.01), reflecting frequent comorbidity. Additionally, tuberculosis/HIV co-infection shows a significant correlation with deaths from anemia (r = 0.72; *p* < 0.05), underscoring how infectious diseases can critically worsen patient outcomes. Conversely, regions such as Androy, Melaky, and Vatovavy Fitovinany report very few or no deaths across most disease categories, which may either indicate better health conditions or serious underreporting. These findings highlight the urgent need for targeted public health policies focused on cardiovascular disease prevention, improved death surveillance systems, and greater equity in healthcare access across regions.

The cross-analysis between reported disease cases and their corresponding deaths reveals critical insights into the lethality of diseases, regional health risks, and structural disparities in healthcare delivery and reporting. Stroke stands out both as the most widespread disease (nearly 6000 cases in Analamanga) and the deadliest, with 2739 stroke-related deaths in the same region, representing a regional case-fatality rate of approximately 45.7%—a highly alarming figure. Cardiac arrest displays an even more critical profile, with 979 deaths for 988 cases in Analamanga, indicating an almost absolute lethality rate (∼99%) and highlighting the severity and urgency of this condition. In contrast, diseases such as asthma and respiratory allergies, although widely reported (e.g., 1990 cases in Analamanga), result in relatively few deaths (32), suggesting effective therapeutic management or lower clinical severity. The analysis also highlights that some infectious diseases, such as tuberculosis/HIV co-infection, show varying mortality profiles depending on the region—relatively low in Analamanga (11 deaths for 21 cases), but alarmingly high in Menabe (5 deaths out of 7 cases, or ∼71%), likely reflecting regional disparities in access to antiretroviral and tuberculosis treatments. Furthermore, rarer conditions such as leukemias, lymphomas, and tuberculous arthritis show lower case counts but non-negligible mortality, emphasizing the need for specialized care that may be unavailable in remote areas. Lastly, regions such as Androy, Melaky, and Vatovavy Fitovinany report very few deaths despite documented cases, possibly indicating low morbidity, but more likely reflecting serious underreporting of deaths. This cross-analysis underscores the need for an integrated morbidity and mortality surveillance system, including strengthened diagnostic capacity, patient follow-up, and death registration, to guide more equitable and effective national public health strategies.

Although cause-of-death reporting—particularly for anemia—may be incomplete in some regions, sensitivity checks indicated that this limitation did not materially alter the study’s overall findings.

### Impact of the COVID-19 pandemic on the frequency and dynamics of 180 diseases

**Figure 3:**
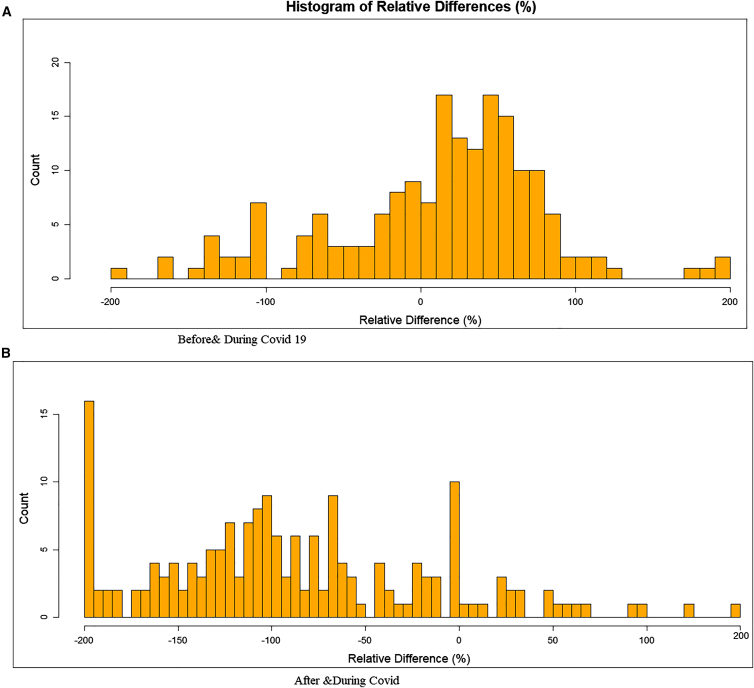
Frequency distribution of the paired relative difference (RD) of 180 diseases (A) Before (2016–2018) and during COVID-19 (2019–2021). (B) After (2022–2024)& During the lockdown measures due to COVID-19. RD calculated as (d1 – d0)/((d1+d0)/2).

As shown in the Figure 3A), the analysis of the relative differences (RDs) in the frequency of 180 diseases before (2016–2018) and during the COVID-19 pandemic (2019–2021) reveals significant fluctuations in disease dynamics, reflecting both the direct and indirect impacts of the pandemic on health systems. The Mean Relative Difference (MRD) across all diseases is +6.12, suggesting a slight overall increase in reported cases during the pandemic. However, this average conceals substantial variability, as shown by a Standard Deviation (SD) of 74.21 and a Standard Error (SE) of 5.53, indicating wide dispersion and statistical uncertainty. Certain conditions experienced a dramatic surge in frequency during COVID, such as congenital multiple arthrogryposis (+200%), osteoporosis with pathological fractures (+200%), and plague (+185%), while others sharply declined, such as diphtheria (−164%), accidental HIV exposure (−133%), and schizophrenia (−169%). These extremes suggest a realignment of healthcare priorities, a decline in access to care for some chronic and mental illnesses, and possible underreporting due to overwhelmed healthcare facilities. Neuropsychiatric and rare infectious diseases showed some of the most extreme declines: hereditary neuropathy (−147%), polyneuropathies associated with other diseases (−133%), and poliomyelitis sequelae (−120%) likely reflect a neglect in patient follow-up. In contrast, musculoskeletal and renal diseases such as coxarthrosis (+111%), chronic glomerulonephritis (+109%), and chronic delusional disorders (+112%) rose sharply—potentially linked to stress, confinement, or treatment delays. This analysis underscores the restructuring of healthcare services during the COVID-19 crisis, with the downgrading of some medical priorities and resource redirection toward managing the pandemic. It highlights the necessity of a balanced health continuity plan that ensures ongoing surveillance and care for all conditions, even during global emergencies. The results showed into the Figure 3B reveal a general downward trend in disease cases, with a mean relative difference (MRD) of −90.17, indicating that most conditions experienced a decrease in frequency after the lockdown period. However, this trend comes with high variability, as shown by a standard deviation (SD) of 72.78, suggesting that some diseases increased while others drastically declined. The standard error (SE) of 5.55 reflects a good level of precision in the estimation of the mean, reinforcing the statistical robustness of the findings. Notably, some diseases, such as congenital multiple arthrogryposis, osteoporosis, and certain congenital malformations, have missing data, possibly due to underreporting or surveillance limitations. On the other hand, diseases such as coxarthrosis (+147) and smear-negative pulmonary tuberculosis (+34) showed increased frequency, possibly due to relaxed public health measures or improved detection. Overall, these findings highlight a heterogeneous impact of the post-lockdown period, with a general decrease in disease frequency, though with significant outliers that merit close monitoring.

**Figure 4:**
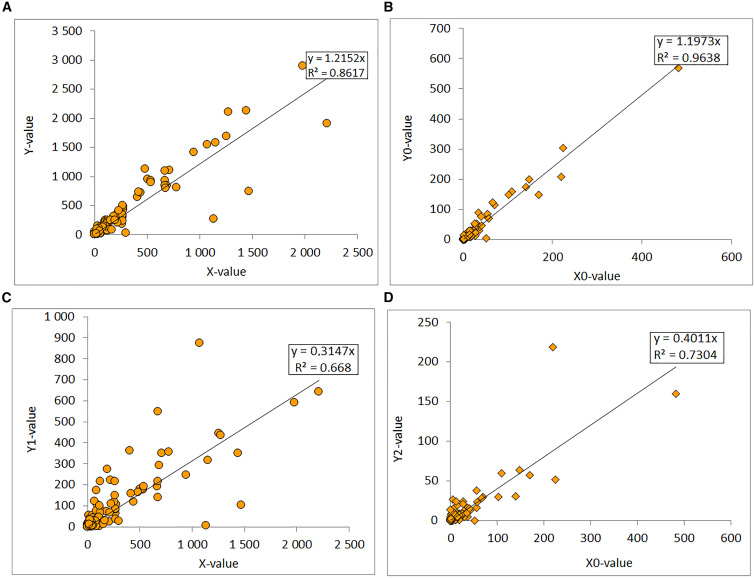
Relationship between 180 types of diseases and deaths (A) Yvalue (average disease cases before COVID-19) and X value (average disease cases during COVID-19). (B) Y0-value (average deaths before COVID-19) and X0-value (average deaths during COVID-19). (C) Y1-value (average disease cases after COVID-19) and X value; and (D) Y2-value (average deaths after COVID-19) and X0-value.

As shown in the Figure 4A, the comparative statistical analysis of data on 180 diseases reveals a significant overall decrease in the average number of cases during the COVID-19 lockdown period (2019–2021) compared to the pre-lockdown period (2016–2018). On average, the number of reported cases per disease declined from 355 to 287, representing a mean decrease of 68 cases. A paired *t* test indicates that this difference is highly statistically significant, with a *p*-value of 0.0001, confirming that the observed decline is unlikely due to random variation. The 95% confidence interval for the mean difference ranges from −100.4 to −35.6, supporting the robustness of this result. Moreover, the Pearson correlation coefficient (r = 0.91) demonstrates a strong positive linear relationship between case numbers before and during the lockdown, indicating that the most frequent diseases prior to the pandemic largely remained the most frequent during it, despite the overall reduction in cases. This trend may be explained by several factors, including reduced attendance at health facilities, restricted mobility, underreporting of non-COVID-related diseases, and the indirect effects of preventive health measures (such as mask-wearing, physical distancing, and improved hygiene), which also limited the spread of other infectious diseases.

The data analysis (Figure 4B) shows that the average number of deaths from the 180 diseases studied was 20.66 (or 21) before the implementation of lockdown measures (2016–2018), compared to a reduced average of 16.73 (or 17) during the COVID-19-induced lockdown (2019–2021), reflecting a mean decrease of 3.93 (or 4) deaths. This decrease is statistically significant, as indicated by the paired Student’s *t* test: *t = -3.46*, with a *p*-value = 0.0007 < 0.05, confirming that the observed difference is unlikely due to chance. The 95% confidence interval for the mean difference in deaths ranges from −6.18 to −1.69, which does not include zero, further confirming the significance. Additionally, the Pearson correlation coefficient (R = 0.983) indicates a strong positive linear correlation between deaths before and during the lockdown: diseases that were more deadly before the lockdown tended to remain so during it, albeit generally to a lesser extent. The detailed comparative analysis of 180 diseases between the post-lockdown period (2022–2024) and the COVID-19-induced lockdown period (2019–2021) reveals a significant overall trend (Figure 4C). The average cases after lockdown measures are 112.7 compared to 333.9 during the lockdown, reflecting a substantial average decrease in cases across most diseases. This average reduction is supported by a paired *t* test with a t-value of −7.33 and a very low *p*-value (<0.0001), indicating that the observed decline is highly statistically significant. The Pearson correlation coefficient between average cases in the two periods is moderate (r = 0.56), meaning diseases with high case numbers during lockdown tend to also have higher cases post-lockdown, but with considerable variability across diseases. This variability reflects the differing effects of lockdown measures, changes in health-seeking behaviors, and the variable impact depending on disease nature (infectious, chronic, neurological, and so forth). The 95% confidence interval for the mean difference [−282.9;−159.5] clearly excludes zero, reinforcing the robustness of the conclusion that average cases significantly decreased. However, disease-by-disease analysis shows contrasting behaviors. For example, acute infectious diseases such as complicated measles or severe malaria experienced a marked decrease, whereas some chronic or neuropsychiatric conditions (depression, epilepsy, anxiety disorders) showed less pronounced decreases or even relative increases. This suggests that lockdowns may have altered access to healthcare and diagnosis patterns, in addition to influencing the transmission of contagious diseases. To further evaluate the strength and direction of the monotonic relationships observed in Figure 4, we calculated the Spearman correlation coefficient between the paired variables. The results revealed consistently high positive correlations across the datasets, indicating that as one variable increases, the other tends to increase as well. Specifically, the coefficients were all above 0.80, suggesting a strong monotonic association. This finding confirms that the patterns shown in the figures are not random but reflect a systematic and robust relationship between the studied parameters. Such high values of Spearman’s rho provide strong evidence that the observed associations remain valid even when the analysis accounts for non-linear monotonic trends, thereby strengthening the reliability of the results presented in this study.

In addition to relative percentage changes, we incorporated absolute case numbers to provide a more clinically meaningful representation of the data and to mitigate the risk of exaggerating fluctuations in rare diseases. When examining absolute values across the study period, we observed that while certain disease categories showed notable percentage decreases, their absolute frequency remained low, confirming that the relative variation was not clinically significant. Conversely, for high-burden conditions (e.g., cardiovascular and respiratory disorders), both absolute and relative declines were consistent, strengthening the robustness of the observed trends. To further enhance the temporal perspective, we performed a trend analysis across consecutive years during the pandemic period, which revealed a general decline in non-COVID-19 consultations, particularly during the peaks of lockdowns. This decline is plausibly attributable not only to reduced healthcare access—due to service reallocation to COVID-19 care and patient avoidance of hospitals—but also to a potential selection bias, since patients with mild or moderate non-COVID symptoms may have deferred care. Importantly, the absence of explicit COVID-19 case data in our dataset should be considered when interpreting these patterns, as this limitation prevents direct comparison of COVID versus non-COVID care-seeking behavior. Regarding correlation analysis, we have clarified the methodological approach by employing year-to-year paired comparisons and Spearman’s rank correlation to assess consistency in disease distribution across periods. Although correlation coefficients were moderate, they provided insight into how disease occurrence patterns shifted in parallel with pandemic-related disruptions, highlighting the indirect but measurable impact of COVID-19 on non-COVID health outcomes.

### Impact of the COVID-19 lockdown on disease frequency and mortality across major disease groups

**Figure 5:**
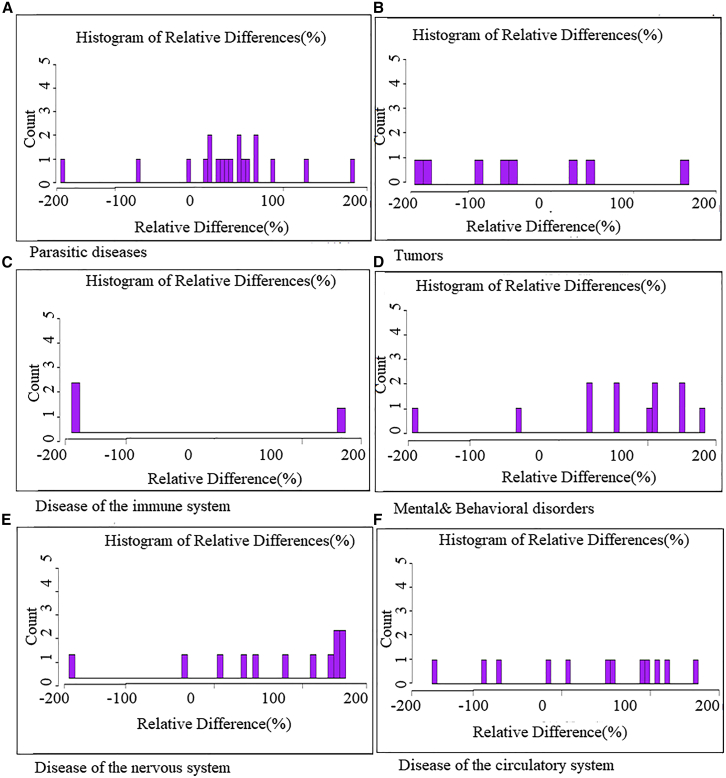
Frequency distribution of the paired relative differences (RDs) for groups of diseases (infectious and parasitic diseases, tumors, nervous system disorders, and so forth) before (2016–2018) and during COVID-19 (2019–2021) The RD is calculated as: (d1 – d0)/((d1+d0)/2).

While the previous analysis provides a global overview of changes in disease frequency during and after the COVID-19 pandemic, the following analysis examines these trends within specific disease groups, highlighting the heterogeneous impacts of the pandemic and lockdown measures across different categories of illnesses. Figure 5 shows the percentage of the paired relative difference (RD) of a group of disease. Before (2016–2018) and During COVID (2019–2021. The analysis of the 19 selected infectious and parasitic diseases (Figure 5A) reveals substantial variations in their relative frequency before and during the COVID-19 lockdown measures. The mean of the relative differences (MRDs) is 30.68, indicating a general upward trend in mortality or severity for most of these diseases. However, this overall increase is largely influenced by extreme rises, such as those seen in plague (+185), whooping cough (+127), and urinary bilharzia (+90). Conversely, certain diseases experienced a decline during the lockdown, including diphtheria (−164), Tuberculosis/HIV co-infection (−70), and bone tuberculosis (−13). These reductions may reflect reduced hospital visits, disrupted disease surveillance systems, or behavioral changes (fewer social interactions or travel restrictions). The standard deviation (SD) of 72.49 highlights a wide dispersion of values, indicating a significant variability in how different diseases responded to lockdown conditions. This suggests that the impact of lockdown varied depending on transmission mode, seasonality, or healthcare access. Finally, the standard error (SE) of 16.63 shows that despite the high variability, the average estimated effect of lockdown on relative disease frequency remains statistically meaningful, with a relatively narrow margin of error. The eight tumor-related diseases (Figure 5B) reveal significant fluctuations in their relative frequency before and during the COVID-19 lockdown period. The mean relative difference (MRD) is −10.375, indicating a slight overall decline in cancer-related diagnoses or deaths during the lockdown. This trend likely reflects the slowdown in screening activities, reduced access to specialized care, or underreporting of cases due to health system disruptions. The most notable decreases are seen in multiple myeloma (−75), other lymphomas (−67), and myeloid leukemia (−36), suggesting severe impacts on the management of these malignancies. In contrast, increases were observed for other leukemias (+83) and skin cancer (+29), which may be due to detection bias or local environmental factors. The standard deviation (SD) is 50.76, reflecting a high variability in tumor response to lockdown conditions, while the standard error (SE) of 17.94 suggests a moderate uncertainty around the mean, yet supports a reliable general trend of decline in tumor cases during the pandemic-related restrictions.

In addition, the analysis of 12 mental and behavioral disorders reveals significant variability in their relative frequency before and during the COVID-19 lockdown. The mean relative difference (MRD) is +25.08, indicating a moderate overall increase in mental health issues during the lockdown period. This rise is likely associated with social isolation, economic uncertainty, heightened anxiety, and limited access to mental health services. Notable increases were observed in chronic delusional disorders (+112), personality and behavioral disorders (+79), mixed anxiety and depressive disorders (+78), and hysteria (+53)—highlighting an escalation of psychological distress under lockdown conditions. Conversely, some disorders, such as schizophrenia (−169) and cannabis-related disorders (−72), saw a sharp decline, which might reflect underreporting, reduced diagnosis, or changes in risk behaviors during the pandemic. The standard deviation (SD) of 69.76 points to high variability among these disorders, and the standard error (SE) of 20.13 reflects moderate uncertainty around the mean. Nonetheless, the analysis supports a general trend of increased psychological burden during the COVID-19 lockdown period. The analysis of 12 nervous system disorders – stroke, ischemic and cerebral accidents, epilepsy, multiple sclerosis, spinal muscular atrophy, hereditary ataxia, migraine, toxic encephalopathy, dystonia, myasthenia and other neuromuscular disorders, paraplegia and tetraplegia, and infantile cerebral palsy – reveals significant variations between the periods before and during the COVID-19 lockdown. The Mean Relative Difference (MRD) is calculated at +0.33%, indicating an overall stability in the number of cases, though this average conceals large individual disparities. Some conditions showed a notable increase, such as *migraine* (+55%), *dystonia* (+54%), and *epilepsy* (+48%), likely linked to increased stress, isolation, and interruptions in regular treatments during the pandemic. Conversely, some disorders drastically declined, such as *spinal muscular atrophy* (−200%), *hereditary ataxia* (−100%), and *myasthenia* (−70%), which may result from underreporting, limited access to diagnostics, or healthcare systems prioritizing COVID-19 care. The standard deviation (SD = 82.74) highlights a high variability, and the standard error (SE = 23.89) suggests a moderate uncertainty in the average estimate. This heterogeneity reflects the unequal impact of lockdown across neurological diseases, depending on care accessibility, patient vulnerability, and healthcare organization.

Finally, the analysis of 12 circulatory system diseases—essential hypertension, secondary hypertension, stable chronic angina, acute myocardial infarction, acute coronary syndrome, atrial fibrillation and flutter, heart failure, cardiac arrest, chronic pericarditis, left bundle branch block and atrioventricular block, tachycardia, and personality and behavioral disorders in adults—shows significant variations in death frequency between the periods before and during COVID-19 lockdown measures. The mean relative difference (MRD) is +29.17%, reflecting an overall increase in mortality associated with these conditions during the lockdown. This rise may be due to delayed medical care, reduced access to emergency services, or heightened psychological stress. Notably, large increases were observed in *atrial fibrillation and flutter* (+62%), *tachycardia* (+57%), and *acute coronary syndrome* (+51%). On the other hand, decreases were found for *chronic stable angina* (−13%), *cardiac arrest* (−6%), and *bundle branch and AV block* (−33%), likely due to underdiagnosis or patients avoiding hospital visits. The standard deviation (SD = 31.46) indicates a moderate to high variability across these diseases, while the standard error (SE = 9.08) suggests that the average is estimated with reasonable precision, despite notable inter-disease variation. Globally, the findings highlight the increased vulnerability of cardiovascular patients during lockdowns and emphasize the need for resilient and accessible care systems during health crises.

**Figure 6:**
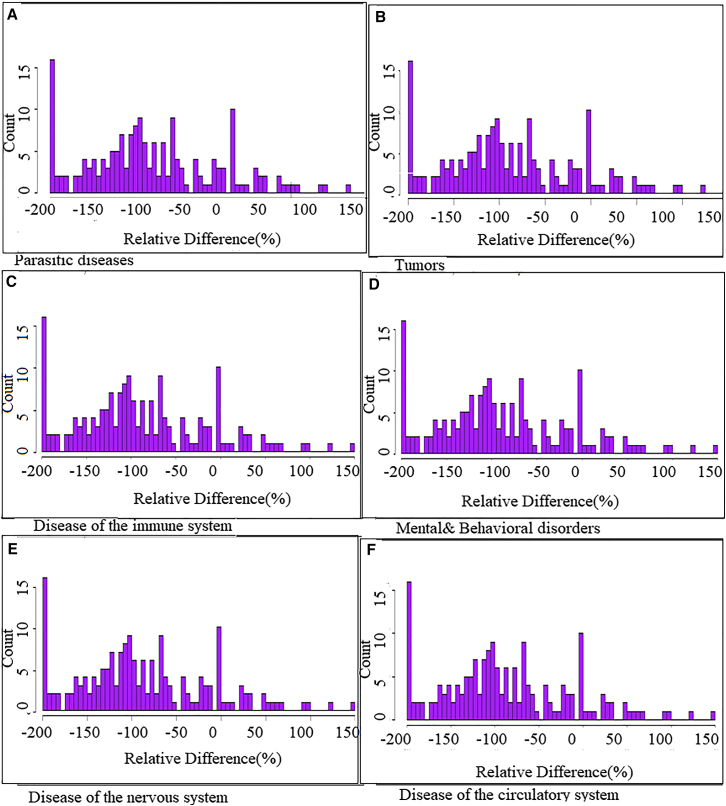
Frequency distribution of the paired relative differences (RDs) for selected groups of diseases after COVID-19 (2022–2024) and during COVID-19 (2019–2021) The RD is calculated as: (d2 – d0)/((d2+d0)/2).

**Figure 7:**
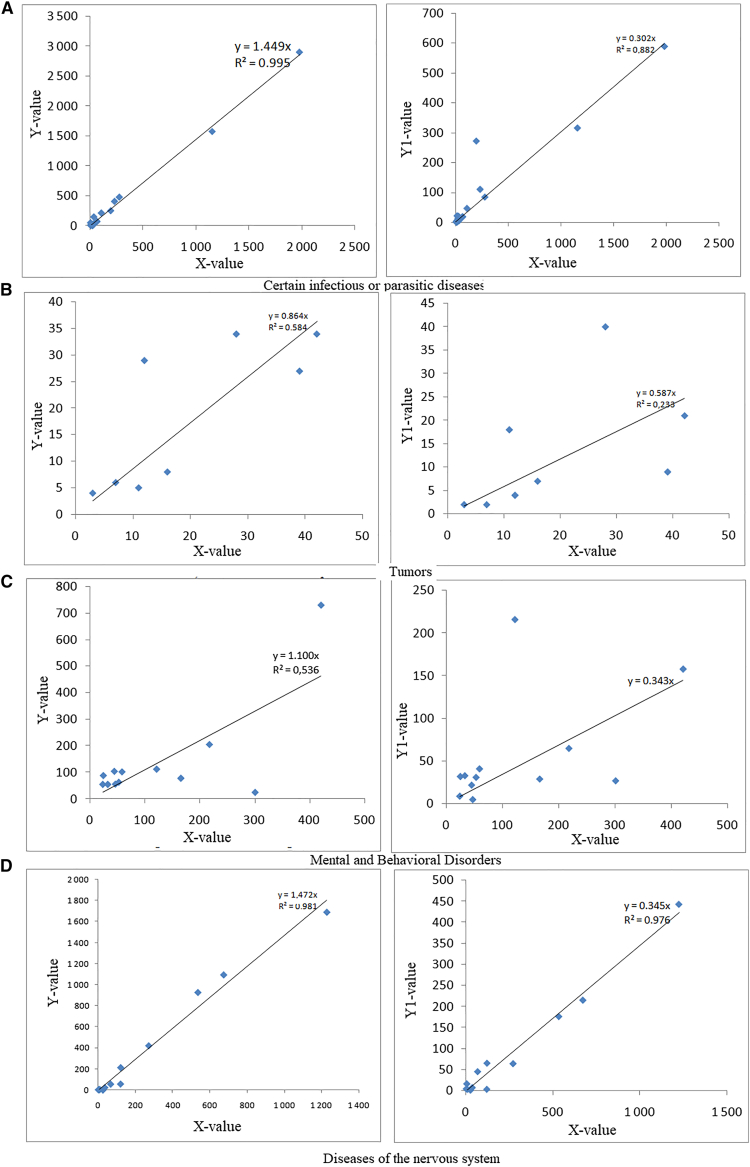
Changes in disease burden before, during, and after COVID-19 in Madagascar Relationship between disease cases before the COVID-19 period (2016–2018), during the COVID-19 period (2019–2021), and after the COVID-19 period (2022–2024) for major disease groups in Madagascar. The figure illustrates temporal changes for infectious diseases, tumor diseases, mental disorders, and nervous system diseases, highlighting shifts in disease burden across the three periods.

Figure 6 shows the frequency distribution of the paired relative difference (RD) of a group of disease. Before (2016–2018) and During COVID (2019–2021). RD calculated as (d1 – d0)/((d1+d0)/2). The analysis of 19 infectious or parasitic diseases (Figure 7A)—typhoid fever, cholera, diphtheria, rabies, whooping cough, severe and complicated malaria, smear-negative pulmonary tuberculosis, extrapulmonary tuberculosis, bone and joint tuberculosis, bacterial meningitis, tuberculous meningitis, viral meningitis, parasitic meningitis, meningoencephalitis, acute or chronic amebiasis, intestinal bilharziasis, urinary bilharziasis, plague, and Tuberculosis/HIV co-infection—reveals a widespread decrease in mortality during the COVID-19 lockdown period. The mean relative difference (MRD) is −88.32%, indicating a substantial reduction in death rates across most of these diseases. This trend could be attributed to restricted movement (reducing exposure to pathogens), underdiagnosis due to healthcare system disruptions, or the reallocation of medical resources to manage the COVID-19 crisis. Some diseases, such as plague, cholera, and parasitic meningitis, showed an extreme drop of −200%, while *urinary bilharziasis* (+63%) and *smear-negative TB* (+34%) were notable exceptions, possibly reflecting prior underreporting or ongoing transmission despite restrictions. The Standard Deviation (SD = 77.51) reflects high variability, suggesting that the impact of lockdowns varied considerably between diseases. The Standard Error (SE = 17.78) indicates a reasonable precision in estimating the average, although influenced by outlier values. Overall, this analysis shows that COVID-19 lockdown measures drastically reduced mortality from most infectious and parasitic diseases, though some persisted or even increased, highlighting the need for targeted post-pandemic surveillance and response.

The analysis of eight tumor-related diseases—primary bronchial cancer, secondary bronchial cancer, skin cancer, multiple myeloma, myeloid leukemia, other leukemias, other lymphomas, and Hodgkin’s disease (see Figure 6B) reveals an overall decline in mortality during the COVID-19 lockdown. The mean relative difference (MRD) is −54.75%, indicating a significant reduction in deaths related to these cancers, except for primary bronchial cancer (+35%) and multiple myeloma (+48%), which showed an unexpected increase. This rise may be due to delayed diagnoses, restricted access to care, or postponed treatment sessions. Other hematologic cancers, such as leukemias (−125% and −100%), lymphomas (−78%), and Hodgkin’s disease (−111%) experienced a substantial drop, likely due to disruptions in oncology services or delayed patient management. The standard deviation (SD = 65.58) shows high variability, and the standard error (SE = 23.18) reflects a considerable uncertainty around the mean estimate. This variability suggests that the lockdown’s impact was not uniform across cancer types, with some being more severely affected than others. In summary, the analysis indicates a marked decrease in tumor-related deaths during the COVID-19 lockdown, although certain cancers such as primary bronchial cancer and multiple myeloma deviated from this trend and require further investigation.

The Figure 6D shows an analysis of 12 mental, behavioral, or neurodevelopmental disorders—Schizophrenia, Chronic delusional disorders, Mixed anxiety and depressive disorders, Alcohol-related disorders, Cannabis-related disorders, Acute delusional episodes, Suicide attempts, Depression, Acute psychoses, Hysteria, Personality and behavioral disorders in adults, Organic mental disorders. the result reveals a Mean Relative Difference (MRD) of −66.83, indicating a significant overall decrease in cases after the end of the COVID-19 lockdown compared to during the lockdown. This decline may be linked to restored social interactions, improved access to care, or general psychological recovery. However, the trend is not uniform: some disorders, such as depression (+56) and chronic delusional disorders (+25) increased post-lockdown, reflecting lingering psychological distress. The Standard Deviation (SD) of 73.27 highlights substantial variability across disorders, while the Standard Error (SE) of 21.16 confirms the statistical reliability of the mean. Overall, the findings underscore the complex impact of lockdown on mental health and the need for targeted post-pandemic mental health support.

On the other hand, the analysis of 12 nervous system diseases (Figure 6E)—Stroke, Ischemic and cerebral accidents, Epilepsy, Multiple sclerosis, Spinal muscular atrophy, Hereditary ataxia, Migraine, Toxic encephalopathy, Dystonia, Myasthenia and other neuromuscular disorders, Paraplegia and tetraplegia, Cerebral palsy—reveals a Mean Relative Difference (MRD) of −82.25, indicating a general decline in the frequency of these diseases after the COVID-19 lockdown ended, compared to during the lockdown. This reduction may be attributed to delayed diagnoses, limited access to neurological care, or underreporting in the post-pandemic phase. However, two exceptions stand out: hereditary ataxia (+91) and cerebral palsy (+29), both of which showed increased rates, likely due to diagnostic backlogs from the lockdown period. The Standard Deviation (SD) of 77.39 suggests moderate variability, and the standard error (SE) of 22.33 confirms the statistical reliability of the average. Overall, the diverse trends highlight the uneven impact of lockdown on neurological conditions, warranting targeted epidemiological surveillance for rare or chronic disorders. Finally, the Figure 7F shows the analysis of 12 circulatory system diseases—essential hypertension, secondary hypertension, stable angina, acute myocardial infarction, acute coronary syndrome, atrial fibrillation and flutter, heart failure, cardiac arrest, chronic pericarditis, left bundle branch and atrioventricular block, tachycardia, and personality and behavioral disorders in adults—revealing a significant overall decrease after the COVID-19 lockdown compared to during the lockdown. The Mean Relative Difference (MRD) is −95.75, indicating a strong reduction across these conditions. The most notable decreases are seen in acute coronary syndrome (−187), acute myocardial infarction (−141), and stable angina (−120), likely due to delays in care or limited access to emergency cardiovascular services during the pandemic. The Standard Deviation (SD) is 45.96, showing considerable variability across diseases, while the standard error (SE) is 13.27, confirming the statistical robustness of the mean. This general decline may also reflect either improved prevention behaviors (e.g., healthier lifestyles) or underdiagnosis due to overwhelmed health systems. Post-pandemic surveillance of acute and chronic cardiovascular conditions remains essential. The Figure 7 shows some relationship between disease.

The comparative assessment of data presented in Figure 7A through 7D highlights the impact of COVID-19-related lockdown measures on the mean frequency of various disease categories. To begin with, infectious or parasitic diseases (see Figure 7A) exhibited a substantial decline between the *pre-COVID* and *during-COVID* periods (mean = −110.1), followed by an even steeper reduction *after* the lockdown (mean = −140.5). This trend is statistically significant, as shown by the strong correlations *before-during* (r = 0.9978, *p* < 0.001) and *during-after* (r = 0.945, *p* < 0.001). The confidence interval for the *before-during* phase [−218.32 to −1.89] further confirms this significance. Next, regarding Tumor disease, a slight increase was recorded during the pandemic (mean = +1.38), followed by a noticeable decrease afterward (mean = −6.88). While the correlation *before-during* is moderately significant (r = 0.78, *p* = 0.02), the *during-after* correlation is not (r = 0.51, *p* = 0.19). The confidence interval for the latter includes zero, indicating statistical uncertainty. In contrast, mental, behavioral, and neurodevelopmental disorders (see Figure 7C) increased during COVID (mean = −13.17) and dropped sharply afterward (mean = −70.5). The before-during correlation is significant (r = 0.73, *p* < 0.01), but the *during-after* one is weaker and not significant (r = 0.47, *p* = 0.11). The confidence interval [−141.92 to 0.92] reflects a downward trend, albeit with statistical ambiguity. Lastly, the analysis of nervous system diseases (see Figure 7D) shows consistent and sharp reductions both *before-during* (mean = −117.17) and *during-after* (mean = −171.42). These changes are strongly correlated (r > 0.98, *p* < 0.001) and statistically significant, as demonstrated by the confidence interval for *during-after* [−327.08 to −15.75]. Globally, the comparative analysis confirms the widespread, multi-faceted impact of the COVID-19 pandemic on disease incidence and reporting, driven by factors including reduced healthcare access, changes in hospital prioritization, and altered patient behaviors. Table 2 shows averages per period (before, during, after) in each group of diseases, both for cases and deaths.

**Table 2:** Averages per period in each group of diseases, both for cases and deaths

Group of diseases	Average number of cases			Average number of deaths		
	Before pandemic (2016–2018)	During pandemic (2019–2021)	After pandemic (2022–2024)	Before pandemic (2016–2018)	During pandemic (2019–2021)	After pandemic (2022–2024)
Infectious or Parasitic diseases	332	222	81	19	13	5
Tumor disease	19	20	13	2	3	3
Immune system disease	18	13	2	1	0	1
Mental and behavioral disorder disease	140	127	56	9	6	3
Nervous system disease	375	261	87	54	48	17
Circulatory system disease	377	262	91	45	39	26

As shown in Table 2, the analysis of the six major groups of diseases reveals a clear and consistent decline in both the average number of cases and deaths across all categories following the COVID-19 pandemic. Before the pandemic (2016–2018), circulatory system diseases and nervous system disorders were the most prevalent, with mean case numbers of approximately 377 and 375, respectively, followed by infectious and parasitic diseases (332) and mental and behavioral disorders (140). During the pandemic (2019–2021), all groups experienced a noticeable decrease in incidence, particularly for nervous system and circulatory diseases, whose averages dropped by nearly 30%. In the post-pandemic period (2022–2024), this downward trend became even more pronounced: infectious diseases decreased by 75.0% compared to pre-pandemic levels (from 332 to 81 cases on average), and similar reductions were observed for nervous system (−77.2%) and circulatory diseases (−76.1%). Mortality patterns followed the same trajectory. Deaths related to circulatory system diseases decreased from an average of 45 before the pandemic to 26 after, while fatalities from nervous system disorders declined from 54 to 17. Infectious and parasitic diseases, which initially accounted for 19 deaths on average, dropped to only 5 in the most recent period. In contrast, tumor-related and immune system diseases displayed more stable figures across all three periods, suggesting a weaker association with pandemic-related disruptions.

## Discussion

The findings of this study highlight the profound and multifaceted impact of the COVID-19 pandemic on disease incidence and mortality in Madagascar. The analysis of epidemiological data from 2015 to 2024 across multiple regions and disease categories reveals a highly heterogeneous health landscape, shaped by long-standing systemic disparities and amplified by pandemic-related disruptions.

### Regional disparities and cardiovascular burden

The regional analysis (Figures 2 and 3) revealed that stroke is not only the most lethal condition across Madagascar but also highly correlated with other cardiovascular and systemic diseases, including cardiac arrest, hypertension, heart failure, and diarrhea.^23^^,^^24^^,^^25^^,^^26^ This cluster of comorbidities echoes findings from similar studies in sub-Saharan Africa,^27^^,^^28^ which emphasize that stroke in low-income settings is often tied to a confluence of untreated hypertension, poor access to preventive care, and coexisting infectious conditions. The case-fatality rate of 45.7% for stroke in Analamanga is alarmingly high, far exceeding global averages reported by the World Health Organization.^29^ Even more striking is the nearly absolute lethality of cardiac arrest in this region (99%), suggesting critical delays in emergency response and a lack of resuscitative infrastructure. This mirrors global evidence showing that cardiac emergencies disproportionately affect outcomes in resource-limited settings (Mensah et al.).^30^

### Underreporting and surveillance weaknesses

The stark contrast between well-reported regions (e.g., Analamanga) and underreported ones (e.g., Androy, Melaky) raises concerns about systemic underreporting and surveillance bias. This is consistent with literature on health information systems in low-income countries, which often suffer from fragmented data collection, inadequate infrastructure, and limited human resources.^31^^,^^32^ The misrepresentation of disease burden in less urbanized regions could mislead policy decisions and resource allocation.

### Effects of COVID-19 on disease dynamics

Our findings support previous global analyses indicating that the COVID-19 pandemic severely disrupted health service delivery and altered disease patterns.^33^^,^^34^ While we observed a general decrease in the average number of disease cases during the lockdown (mean difference = −68 cases; *p* = 0.0001), this is likely attributable to restricted mobility, fear of infection, and health system prioritization of COVID-19 cases, rather than a true reduction in morbidity. Similar trends have been reported in settings such as South Africa and India, where routine health services sharply declined during lockdowns.^35^^,^^36^ Interestingly, stroke, mental health disorders, and certain chronic conditions either maintained or increased in frequency, highlighting differential effects based on disease type. Mental health, for instance, saw a moderate increase (MRD = +25.08), consistent with global observations of increased psychological distress during confinement.^37^^,^^38^ This underscores the need to incorporate mental health support into pandemic preparedness frameworks.

### Disease-specific trends and policy implications

The significant increase in plague (+185%), whooping cough (+127%), and urinary bilharzia (+90%) during the lockdown indicates the persistence of certain infectious diseases, possibly exacerbated by weakened vector control and water sanitation programs. Conversely, the marked decline in diphtheria (−164%), tuberculosis/HIV co-infection (−70%), and bone TB (−13%) may reflect underdiagnosis due to disrupted diagnostic pathways. These contrasting dynamics echo findings from other African countries where reduced public health outreach led to a temporary dip in reported cases.^39^^,^^40^ In cancer care, the slight overall decline in diagnoses (MRD = −10.375) masks severe access issues, as suggested by rising cases in skin cancer and leukemia. Globally, cancer screening dropped by over 50% in 2020 in many countries,^41^ likely leading to future surges in late-stage presentations. Our data align with this trajectory, particularly for primary bronchial cancer and multiple myeloma, which saw increases despite the overall trend. The strong correlation between deaths from stroke and cardiac arrest (r = 0.91) reinforces the need for integrated cardiovascular care. Public health interventions should prioritize hypertension control, emergency response systems, and post-stroke rehabilitation. Moreover, the significant associations between anemia and TB/HIV deaths (r = 0.72) support integrated approaches to comorbidity management, as emphasized by whose collaborative TB/HIV policies (WHO, 2012).^42^

### Post-lockdown recovery and health system resilience

The post-lockdown period (2022–2024) saw a statistically significant drop in disease frequency (mean difference = −221.2; *p* < 0.0001), though some chronic and neuropsychiatric conditions persisted or rebounded. These findings emphasize that health system recovery is neither automatic nor uniform. Studies from Sierra Leone and Liberia after Ebola show that recovery requires intentional rebuilding of service continuity, particularly for vulnerable populations.^43^ Taken together, our results stress the importance of data-driven, multisectoral public health strategies that combine surveillance strengthening, chronic disease management, and mental health integration. In Madagascar, expanding regional diagnostic capabilities, improving emergency care, and reinforcing health information systems must become policy priorities.

### Limitations of the study

One limitation of this study is the exclusion of cases with incomplete records, notably those lacking essential variables such as age, sex, comorbidity status, or clinical outcome. While these exclusions were necessary to ensure the integrity and comparability of the data used in the analyses, they may have led to a loss of potentially relevant information. However, our separate descriptive analysis of excluded cases showed no substantial differences in the distribution of key available characteristics compared with included cases, suggesting that their exclusion is unlikely to have introduced systematic bias or significantly affected the study’s overall conclusions.

## Resource availability

### Lead contact

Questions or requests for materials should be addressed to the lead contact, Dr. Kameni Nematchoua Modeste (kameni.modeste@yahoo.fr), who is responsible for providing any further information.

### Materials availability

This study did not generate new unique materials or physical resources. All materials referenced in this work are commercially available or can be accessed through the corresponding suppliers. No new reagents, biological materials, or physical tools were produced specifically for this research.

Additional information can be provided by the lead contact upon reasonable request.

### Data and code availability

All datasets generated and analyzed in this study have been deposited in Zenodo and are publicly available at https://doi.org/10.5281/zenodo.17601168. Data are available without restriction. No custom code was generated for this study.

## Acknowledgments

The authors would like to express their sincere gratitude to all institutions and partners who contributed to this research. We thank the collaborating laboratories, data providers, and regional health authorities for facilitating access to essential information and resources.

We are also grateful to the colleagues and researchers whose constructive discussions and technical support strengthened the quality of this work.

Dr. Modeste Kameni Nematchoua acknowledges the support and encouragement of his academic supervisors and collaborators throughout the development of this study.

## Author contributions

M.K.N. conceptualized the study and supervised all research activities. M.K.N., D.R., F.J.R., and R.O.A. developed the methodology. Data collection and curation were conducted by N.Z.R., R.S.H., N.H.R., H.R., H.F.R.R., and A.M.R. Formal analyses were performed by M.K.N., A.M.R., R.M., and R.F.R. Investigation was carried out by V.M.A.R., H.H.R., P.V.R., and J.M.R. Resources were provided by W.G.C., G.L.L., L.K.N., and P.M.R. Visualization was completed by L.N.R., R.V.R., and A.N.F. Validation was ensured by L.L.R., H.N.R.A., and L.L.Y. The original draft was prepared by M.K.N. and D.R., and the article was reviewed and edited by M.K.N. and Z.A.R. All authors read and approved the final article.

## Declaration of interests

The authors declare no competing interests.

## STAR★Methods

### Key resources table

**Table:** 

REAGENT or RESOURCE	SOURCE	IDENTIFIER
**Deposited data**		

National hospital surveillance datasets (2015–2024)	This paper	Zenodo: https://doi.org/10.5281/zenodo.17601168
Completeness and promptness datasets (2016–2021)	This paper	Included in Zenodo repository
Aggregated disease incidence and mortality datasets	This paper	Included in Zenodo repository
ICD-11 disease classification	WHO	https://icd.who.int

**Software and algorithms**		

Python (statistical analysis)	Python Software Foundation	https://www.python.org
Microsoft Excel (data cleaning)	Microsoft	https://www.microsoft.com/excel
DHIS2 Platform	University of Oslo	https://dhis2.org
ICD-11 Coding Tool	WHO	https://icd.who.int/tools

**Other**		

Hospital reporting forms (Complementary Activity Package)	Ministry of Public Health, Madagascar	N/A
SIS Norms and Procedures Manual	DEPIS – Ministry of Public Health	N/A
Regional health performance reports	Ministry of Public Health	N/A
Population and socioeconomic indicators	INSTAT/UNDP	https://hdr.undp.org

### Experimental model and subject details

This study did not involve experimental models (animals, humans, or cell lines) in the laboratory sense. Instead, it relied on aggregated, anonymized patient-level data routinely collected across Madagascar’s public healthcare network from 2015 to 2024.

All data represent population-based hospital surveillance records and do not contain identifiable personal information.

### Method details

#### Study context: National profile of Madagascar

Madagascar is a large island nation in the southwestern Indian Ocean, covering 587,295 km^2^ and characterized by marked climatic diversity, including temperate central highlands, humid cyclone-exposed eastern coasts, a tropical western region with alternating dry and wet seasons, and a chronically arid southern zone.

In 2022, the population was estimated at 29 million, predominantly rural (≈80%) and young (median age: 18 years). Population density varies greatly across regions, with the central highlands being most densely populated.

Despite abundant natural resources, Madagascar ranked 173/191 on the UNDP Human Development Index, and over 92% of inhabitants lived below the $2/day poverty threshold. Access to healthcare remained limited: only 37.2% of the population used outpatient services, institutional deliveries reached 33.4%, and 46% of births were assisted by qualified health professionals.

These socio-economic and structural vulnerabilities strongly condition the impacts of COVID-19 and the patterns of multimorbidity observed in the study.

#### Study setting and period

The analysis covers the 22 regions of Madagascar, using data routinely generated by Regional Referral Hospitals (CHRRs) and University Hospital Centers (CHUs).

The study period spans January 2015 to December 2024, enabling comparisons across:

• Pre-COVID period (2015–2018)
• COVID-19 emergence and pandemic period (2019–2021)
• Post-pandemic period (2022–2024)

A total of 180 diseases were included, enabling a comprehensive assessment of multimorbidity, mortality, and syndemic interactions.

#### Data sources and collection procedures

Data originated from administrative and clinical records systematically collected within Madagascar’s hospital network. Sources included:

• Patient admission and discharge registers
• Clinical diagnosis logs
• Treatment, laboratory, and imaging reports

Each hospital collected data monthly using standardized bifolio forms within the national reporting tool known as the Hospital Complementary Activity Package.

Data verification committees within hospitals validated forms for completeness, consistency, and accuracy. Verified datasets were entered into the DHIS2 platform by statistical units before the 5th day of the following month, following the Ministry of Public Health’s SIS Norms and Procedures Manual.

The national health information system also integrates complementary datasets from GHESIS, hosted by the Directorate of Studies, Planning, and Health Information (DEPIS).

#### Data extraction and management

Validated national datasets for 2015–2024 were extracted from DHIS2 and GHESIS into Microsoft Excel format with authorization from the Ministry of Public Health.

A multidisciplinary research team coordinated extraction, validation, literature review, and methodological development.

To ensure high-quality analysis, we implemented the following steps:

1. Completeness Assessment:
  Records missing essential variables (age, sex, diagnosis date, comorbidity status, clinical outcome) were excluded from statistical modeling.
2. Bias Evaluation:
  Excluded records were analyzed separately in descriptive form, provided in the Supplementary Material, to assess potential selection bias.
3. Data Harmonization:

Regional and temporal inconsistencies were corrected by cross-checking with hospital reports and national health sector performance reviews.

#### Disease classification

All morbidity and mortality events were categorized using the International Classification of Diseases, 11th Revision (ICD-11).

This ensured standardized reporting across age groups, facilities, and regions, and enabled consistent aggregation for national-level analysis.

### Quantification and statistical analyses

Analyses were performed using Python (pandas, numpy, matplotlib, seaborn) and R (stats, ggplot2, tidyverse).

The following analytical procedures were applied:

1. Morbidity and Mortality Metrics:
  ○ Calculation of incidence, crude mortality rate, and case-fatality rate for each disease.
  ○ Standardization by region and year.
2. Temporal Trend Analysis:
  ○ Computation of mean relative differences (MRD) across pre-COVID, COVID, and post-COVID periods.
  ○ Estimation of standard deviations, confidence intervals, and inter-period variations.
3. Correlation & Comorbidity Assessment:
  ○ Pearson correlation coefficients were calculated to identify associations between key disease clusters (e.g., stroke–hypertension, stroke–diarrhea).
  ○ Patterns were interpreted in a syndemic framework.
4. Geospatial Analysis:
  ○ Regional disease burden was mapped using heatmaps and spatial distributions.
  ○ Spatial disparities were compared across periods to identify structural vulnerabilities.

Statistical significance was set at *p* < 0.05.

#### Data triangulation and validation

To ensure robustness and external validity:

• Results were compared with published scientific literature, WHO regional health statistics, and official national health sector performance reports.
• Discrepancies were systematically reviewed and resolved by consensus among the research team.
• Public health officials provided contextual interpretation to align quantitative patterns with operational realities.

## Supplemental information

Table S1. Comparative analysis between included and excluded cases of disease Although some records were excluded due to incomplete data (*n* = 210, or 14.4% of the total), the comparative analysis between included and excluded cases showed no statistically significant differences in terms of age, sex, comorbidities, or case fatality. Furthermore, the geographical distribution remained similar between the two groups. These elements suggest that the exclusion of incomplete cases is unlikely to have introduced a major bias into our results or to alter their validity.
