# Supplementary material for: Multimorbidity and mortality trends in the COVID19 syndemic in Madagascar covering 180 diseases 2015 2024
Source: iScience. 2025 Dec 17;29(2):114444. doi: 10.1016/j.isci.2025.114444 (PMC12874437; doi:10.1016/j.isci.2025.114444)
Supplement: Table S1. Comparative analysis between included and excluded cases of disease — Although some records were excluded due to incomplete data (n = 210, or 14.4% of the total), the comparative analysis between included and excluded cases showed no statistically significant differences in terms of age, sex, comorbidities, or case fatality. Furthermore, the geographical distribution remained similar between the two groups. These elements suggest that the exclusion of incomplete cases is unlikely to have introduced a major bias into our results or to alter their validity. [file mmc1.pdf]

iScience, Volume *29*

Supplemental information

Multimorbidity and mortality trends

in the COVID19 syndemic in Madagascar

covering 180 diseases 2015 2024

Modeste Kameni Nematchoua, Diana Ratsiambakaina, Faratiana Jenny Rasoariseheno, Rija Onintsoa Andriamasinoro, Nivoarimelina Zoly Rakotomalala, Razafindramboho Samoelà Hérédia, Nirina Henintsoa Raveloharimino, Herisitraka Raotoson, Harimbola Fiononantsoa Razaiarilala Rakotovazaha, Alphonsine Mboty Reziky, Rakotoson Mariette, Raphaël Fidelis Randrianarivo, Vohangy Marie Anita Randriamihaja, Hery Henintsoa Randrianirina, Pâquerette Voahirantsoa Razanamiarana, Joseph Michel Razafimahenina, Wabo Gilles Cédric, Gaël Lauricia Lalanirina, Lindsay Kouatie Njonger, Philippe Manjakasoa Randriantsoa, Luc Narda Randrianahasina, Rakotomalala Vololoniana Razafimanalina, Assoumacou Noro Flavia, Larissa Lalatina Randriamialy, Andriarimanana Hery Nirina Rakotoarisoa, Lethicia Lydia Yasmine, and Zely Arivelo Randriamanantany

Supplemental table

Table3. other data

Variable	Included cases (n = 801,250)	Excluded cases (n = 210)	p-value
Mean age (± SD)	36.8 ± 15.4	37.2 ± 14.9	0.78
Median age (IQR)	35 (24–48)	36 (25–49)	0.81
Male sex (%)	53.1%	52.4%	0.89
Known comorbidity (%)	18.7%	19.3%	0.84
Deaths (%)	4.6%	4.8%	0.92
Geographic region (%)	Proportionally distributed across all 22 regions	Proportionally distributed across all 22 regions	—
